# Supplementary material for: Comparative multiomics analysis of cell physiological state after culture in a basket bioreactor
Source: Sci Rep. 2022 Nov 23;12:20161. doi: 10.1038/s41598-022-24687-4 (PMC9686226; doi:10.1038/s41598-022-24687-4)
Supplement: Supplementary file 1 — Supplementary Information 1. [file 41598_2022_24687_MOESM1_ESM.zip › raw data/Transcriptomic raw data/6.Enrichment/2.KEGG/TE1vsTC1/UP/src/csab05146.html]

csab05146


- csab:103226390

- Up regulated genes

103226390(2.68260263702081)

- csab:103214795

- Up regulated genes

103214795(1.23734090959605)

- csab:103214795

- Up regulated genes

103214795(1.23734090959605)

Close
